# The Genetic Architecture of Vitamin D Deficiency among an Elderly Lebanese Middle Eastern Population: An Exome-Wide Association Study

**DOI:** 10.3390/nu15143216

**Published:** 2023-07-20

**Authors:** Nagham Nafiz Hendi, Marlene Chakhtoura, Yasser Al-Sarraj, Dania Saleh Basha, Omar Albagha, Ghada El-Hajj Fuleihan, Georges Nemer

**Affiliations:** 1 Division of Biological and Biomedical Sciences, College of Health and Life Sciences, Hamad Bin Khalifa University, Doha P.O. Box 34110, Qatar; nhindi@hbku.edu.qa; 2 Calcium Metabolism & Osteoporosis Program, American University of Beirut Medical Center, Beirut P.O. Box 11-0236, Lebanon; mc39@aub.edu.lb (M.C.); dms29@mail.aub.edu (D.S.B.); gf01@aub.edu.lb (G.E.-H.F.); 3 Qatar Genome Program (QGP), Qatar Foundation Research, Development and Innovation, Qatar Foundation (QF), Doha P.O. Box 5825, Qatar; yalsarraj@qf.org.qa; 4 Division of Genomics and Translational Biomedicine, College of Health and Life Sciences, Hamad Bin Khalifa University, Doha P.O. Box 34110, Qatar; oalbagha@hbku.edu.qa; 5 Department of Biochemistry and Molecular Genetics, American University of Beirut, Beirut P.O. Box 11-0236, Lebanon

**Keywords:** exome-wide association study, vitamin D deficiency, genetic determinants, polygenic risk score, Middle Eastern population

## Abstract

The Middle East region experiences a high prevalence of vitamin D deficiency, yet most genetic studies on vitamin D have focused on European populations. Furthermore, there is a lack of research on the genomic risk factors affecting elderly people, who are more susceptible to health burdens. We investigated the genetic determinants of 25-hydroxyvitamin D concentrations in elderly Lebanese individuals (*n* = 199) through a whole-exome-based genome-wide association study. Novel genomic loci displaying suggestive evidence of association with 25-hydroxyvitamin D levels were identified in our study, including rs141064014 in the *MGAM* (*p*-value of 4.40 × 10^−6^) and rs7036592 in *PHF2* (*p*-value of 8.43 × 10^−6^). A meta-analysis of the Lebanese data and the largest European genome-wide association study confirmed consistency replication of numerous variants, including rs2725405 in *SLC38A10* (*p*-value of 3.73 × 10^−8^). Although the polygenic risk score model derived from European populations exhibited lower performance than European estimations, it still effectively predicted vitamin D deficiency among our cohort. Our discoveries offer novel perspectives on the genetic mechanisms underlying vitamin D deficiency among elderly Middle Eastern populations, facilitating the development of personalized approaches for more effective management of vitamin D deficiency. Additionally, we demonstrated that whole-exome-based genome-wide association study is an effective method for identifying genetic components associated with phenotypes.

## 1. Introduction

Vitamin D is an essential nutrient that maintains bone and overall health. A deficiency of vitamin D is widely prevalent worldwide, which is identified when serum concentrations of 25-hydroxyVitamin D (25(OH)D) fall below 50 nmol/L (20 ng/mL). This deficiency has been associated with musculoskeletal disorders, like osteoporosis and rickets in children, as well as cardiovascular diseases and cancer [1,2]. Vulnerable populations, particularly the elderly and obese individuals, are at a higher risk due to reduced sunlight exposure, inadequate intake of vitamin D-rich foods, and intestinal malabsorption [3]. To address this issue, several studies have explored the effectiveness of vitamin D supplementation in managing this deficiency [4]. Additionally, emerging evidence suggests that vitamin D supplementation may have a beneficial effect on weight loss outcomes, potentially through its interaction with receptors involved in energy metabolism [5]. These findings highlight the potential influence of vitamin D on vitamin D deficiency and its associated health issues, underscoring the need for further investigations in this area. However, genetic variations can influence an individual’s predisposition to vitamin D deficiency, vitamin D biosynthesis, and their response to vitamin D supplementation [6,7,8].

Genetic studies have reported heritability estimates of genetic variations in 25(OH)D levels around 80%, underscoring the substantial impact of genetic markers on variations among individuals [9]. Through genome-wide association studies (GWAS), specific genetic variants correlated with 25(OH)D concentrations have been detected in genes responsible for the synthesis and transportation of vitamin D. Notable genes include *CYP2R1* (Cytochrome P450 Family 2R1), *CYP24A1* (Cytochrome P450 Family 24A1), *DHCR7* (7-Dehydrocholesterol Reductase), and *GC* (Vitamin D Binding Protein) [10,11,12,13]. Furthermore, studies have also determined a contribution of genetic variants, including *CYP2R1* and *DHCR7,* to the variability in response to vitamin D supplements [14].

Despite the abundant sunshine in the Middle East, vitamin D deficiency is surprisingly widespread, with rates reaching up to 74% [15,16], and approximately 63% in Lebanon [17]. However, studies investigating the genetic aspects of vitamin D have primarily cantered on individuals of European descent, leaving a knowledge gap in the impact of genetic variables in Middle Eastern populations. We performed the first whole-exome-based GWAS (ExWAS) in elderly Middle Eastern individuals for vitamin D. The ExWAS approach provides an enhanced ability to detect rare variants within protein-coding genes, thus enabling a more effective analysis of the genetic architecture underlying vitamin D deficiency in our population [18]. In order to uncover additional novel and prevalent genetic factors influencing 25(OH)D levels, we conducted a meta-analysis of our cohort with the largest European GWAS (*n* = 417,580 individuals) [11]. Additionally, we assessed the effectiveness of polygenic risk scores (PRS) derived from Europeans along with the correlation of genetic factors and vitamin D deficiency in the elderly Lebanese individuals.

## 2. Methods

### 2.1. Participants’ Characteristics

The present ExWAS study utilized data from three healthcare centers in Lebanon: Rafic Hariri University Hospital, the American University of Beirut-Medical Center, and Hotel Dieu de France. A total of 199 participants aged 65 years or older, with a body mass index (BMI) between 25 and 29 kg/m^2^, were enrolled in this cross-sectional study. Participants with 25(OH)D concentrations between the validated value (10–30 ng/mL) were included. This study, conducted in Beirut, Lebanon from 2011 to 2013, was a randomized controlled trial that evaluated the impacts of two different vitamin D doses on indices of bone mineral density (NCT01315366) [3]. The study received approval from the Institutional Review Board of the American University of Beirut, Lebanon (IM-GEHF-20) on 24 January 2011, and informed written consent was obtained from all participants prior to participation. The identification code assigned to the clinical trial on ClinicalTrials.gov is NCT01315366.

### 2.2. Quantification of 25(OH)D and Related Covariates

Physical measurements, like height and body weight, were taken for all participants by the Seca 284 stadiometer and balance. BMI was calculated by dividing the weight (kg) by the square of height (m^2^). Additionally, a standardized questionnaire was administered to gather information regarding lifestyle factors and medical history. The concentrations of 25(OH)D were assessed with liquid chromatography mass spectroscopy at Mayo Clinic Laboratories in Rochester, MN, USA. Prior to the statistical analyses, the 25(OH)D concentrations for all individuals underwent a rank-based inverse normal transformation using R version 4.1.3. This method entails arranging the values in ascending order, and subsequently, converting the ranks to a normal distribution by utilizing the inverse of the cumulative distribution function. The purpose of this approach is to account for outliers and skewness present in traits that deviate from a normal distribution [19].

### 2.3. Whole-Exome Sequencing and Bioinformatics Analyses

Peripheral blood cells were collected from participants through venipuncture during similar seasons of the year and processed for DNA extraction using a DNA extraction kit (the Qiagen QIAamp blood midi kit, catalog number: 51,185) according to the manufacturer’s guidelines. The resulting samples were assessed for quantity by a NanoDrop (Thermo) at the molecular core facilities of the American University of Beirut, and subsequently stored at a temperature of −80 degrees Celsius. Subsequently, genomic DNA of the 199 samples were transferred to the Macrogen Inc. (Seoul, Republic of Korea) to create DNA sequencing libraries using 101-base-pair paired-end reads on the SureSelectXT Library Prep Kit on Illumina HiSeq 4000 platform. Each sample yielded paired-end reads with a range of 68,807,342–91,844,864 reads and a total of 6.9–9.2 G base-pair reads. Of these, 95.92–96.67% of the reads passed Q30 (a phred quality score of over 30).

Sample-specific FASTQ files, representing the HiSeq reads for that sample, were aligned with BWA MEM to the GRCh37 reference genome [20]. Metric statistics were captured for all samples to evaluate genome capture, variant alignment, and calling quality using SnpEff version 4.2 [21], bcftools [22] with dbsnp version 138 [23], and ClinVar as databases [24]. SNV genotypes with read depth less than 20 and mapping quality less than 40 were excluded. After the DP genotype filtering, duplicate reads followed by SNVs and InDels genotypes were removed using SureCall 2.0 SNPPET algorithm.

To record variant calling for Lebanese samples, we utilized the HaplotypeCaller from GATK (Genome Analysis Toolkit version 3.4, https://software.broadinstitute.org/gatk/documentation/article?id=3238, accessed on 1 February 2023). All the individual intermediate genomic variant call files (gVCF) were used in a joint calling process to create a joint multisample VCF file for all the samples. This process had two steps. In the joint calling process, all sample intermediate genomic variant call files (gVCF) were merged to create a joint multisample VCF file through a two-step process. First, the regions for all the samples were combined using GenomicsDB. Then, GenotypeGVCFs was used to merge all regions, while also applying SNP/Indel recalibration. After applying the VQSR filtration from GATK, only variants that met the specified criteria were retained for further analysis.

We performed a thorough quality control examination via PLINK (version 2.0) to ensure the reliability and integrity of the data [25]. We excluded SNPs that had a MAF < 1%, genotyping call rate < 90%, and Hardy–Weinberg equilibrium *p* < 1 × 10^−6^. Furthermore, we eliminated individuals that had a call rate below 95% (*n* = 5) and conducted checks for gender ambiguity, excess heterozygosity, and duplicates. Multidimensional scaling analysis was also performed using PLINK to detect individuals who deviated from the expected population ancestry. We generated a matrix of pairwise identities-by-state (IBS) using a pruned set of independent autosomal variants, 14,893 SNPs, a window size of 200 variants, and an LD threshold of r^2^ = 0.05 to study the genetic relatedness among the participants in our study. Outliers of population were removed when deviated from the average by four standard deviation units or more (±4 SD) of the first two mds components. A dataset comprising 481,395 SNPs of 194 participants was used on the genome-wide association analysis.

### 2.4. Exome-Wide Association Analysis

We conducted an ExWAS to investigate the relationship between each genetic marker and concentrations of 25(OH)D. We utilized the generalized mixed model implemented in SAIGE [26], which is a linear method based on variance components. This approach corrects for genetic substructure and relatedness by incorporating the genomic kinship matrix. To account for population mixtures, we analyzed the population principal component (PC) through PLINK [25]. The first four principal components (PCs) were included as covariates in the regression model to adjust for population structure along with the adjustment of age and gender. The finding of interest cutoff was set at *p*-value < 5 × 10^−5^ with suggestive significance level of *p*-value below 0.05. R was utilized to create the Manhattan plot and quantile–quantile plot, and to calculate the genomic inflation factor. The linear model of SAIGE was employed to estimate the heritability, quantifying the proportion of variation in levels of 25(OH)D attributed to genetic differences among samples.

The pairwise linkage disequilibrium (LD) among top-associated SNPs was examined using the LD clumping analysis of PLINK software (version 1.9) [25]. We applied an r^2^ threshold of 0.2 within a window of 250 kb to identify SNPs exhibiting strong LD through our ExWAS findings. Subsequently, we utilized LocusZoom software [27] to visualize the LD patterns, which generated regional plots based on the SAIGE summary statistics. These plots effectively highlighted clusters of SNPs demonstrating high LD.

### 2.5. Meta-Analysis

A meta-analysis for the suggestively associated loci was performed by combining the results of our Lebanese ExWAS and a recent large GWAS (GCST90000616) by Revez JA et al. from the UK Biobank (*n* = 417,580 European ancestry individuals) [12]. The GWAS model of GCST90000616 for vitamin D was conducted using similar methods of our analysis, with correction of genotype PCs, age, and gender [12]. The NHGRI-EBI GWAS Catalog [28] provided summary statistics for study GCST90000616 taken in December 2022. The alleles (A1 and A2) in our dataset were compared and validated against the alternate and reference alleles in the referenced dataset (GCST90000616). The statistics of association in our study were canonicalized following the alternate allele as specified in the reference genome. Additionally, both studies used rank-based transformation to inversely normalize 25(OH)D measurements. We performed a meta-analysis using the inverse-variance weighted method and assessed the heterogeneity using PLINK (version 1.9) [25].

### 2.6. Validation of Replicated Loci Previously Associated with 25(OH)D

Our ExWAS results were compared to the European GWAS study for 25(OH)D levels (GCST90000616) [12], to validate the variant replication and correlation of our findings in terms of allele effect value and frequency. We examined the presence of SNPs identified in the meta-analysis within the GWAS catalog (EFO_0004631) for 25(OH)D levels, using the November 2022 release [28]. Our focus was on SNPs that demonstrated significance levels below *p*-value < 5.0 × 10^−8^. Initially, we looked for the genetic regions linked to vitamin D in our cohort and compared it to those present in the UK Biobank dataset. Markers in a region of 250 kb surrounding the GWAS catalog signals were examined, to detect potential novel variants as well as replicated associations with vitamin D.

### 2.7. Analysis of Polygenic Risk Scores

We assessed the performance of a polygenic risk score (PRS) derived from a European population in estimating the genetic predisposition for 25(OH)D levels in the Lebanese population by PLINK (version 1.9) [25]. The PRS was computed by aggregating SNPs based on their risk allele frequency, using the effect weights estimated from GWAS. We obtained the PRS files from the most comprehensive vitamin D GWAS in Europeans (PGS000882: *n* = 417,580 individuals and 1,094,650 variants) [12], through the Polygenic Score Catalog (https://www.PGSCatalog.org, accessed on 15 January 2023) [29].

We then calculated Pearson’s correlation (R) of the PRS derived from Europeans and baseline levels of 25(OH)D, adjusted for the first four principal components, gender, and age through R software. The performance of the derived polygenic score model in detecting individuals having vitamin D deficiency, characterized by concentrations of 25(OH)D less than 20 ng/mL, was assessed through the area under the receiver operating characteristic (ROC) curve (AUC). The range of AUC is from 0.5 (no distinction) to 1 (complete distinction), providing a measure of the PRS model in effectively detecting individuals who are at risk of vitamin D deficiency.

### 2.8. Functional Insights and Annotation of Variants

The annotation of variants associated with 25(OH)D levels were performed using the Ensembl Variant Effect Predictor release 108 (VEP, https://grch37.ensembl.org/index.html, accessed on 15 March 2023) [30]. We utilized the gnomAD (Genome Aggregation Database, https://gnomad.broadinstitute.org, accessed on 16 March 2023) and the ALFA (Allele Frequency Aggregator, www.ncbi.nlm.nih.gov/snp/docs/gsr/alfa/, accessed on 16 March 2023) to compare the frequencies of variants detected in our population to those in global populations. Expressions of quantitative trait loci (eQTLs) of that are associated with the expression levels of novel genes in healthy human tissues were analyzed by ARCHS4 (RNA-seq and ChIP-seq sample and signature search [31]) and GTEx (genotype-tissue expression, http://commonfund.nih.gov/GTEx/, accessed on 18 March 2023, ARCHS4) database.

## 3. Results

### 3.1. Study Description

This study utilizes whole-exome sequence data from elderly Lebanese participants, with an average age of 71 (±4.8) years (interquartile range: 65 to 91 years) and 46.4% female participants (*n* = 104). Notably, the mean of 25(OH)D baseline levels was 20.12 (±7.2) ng/mL (interquartile range: 6 to 44 ng/mL). The average of BMI (in kg/m^2^) was almost equivalent between males and females, with a value of 30.2 ± 4.6 (Table 1). No significant linkages were observed between the 25(OH)D levels and related covariates, such as age, gender, or BMI. Participant enrolment was distributed across all seasons. At enrolment, 19% of participants were on calcium supplementation, and only 0.5% were receiving vitamin D supplements.

### 3.2. Exome-Wide Association Study on 25(OH)D

**Figure 1:**
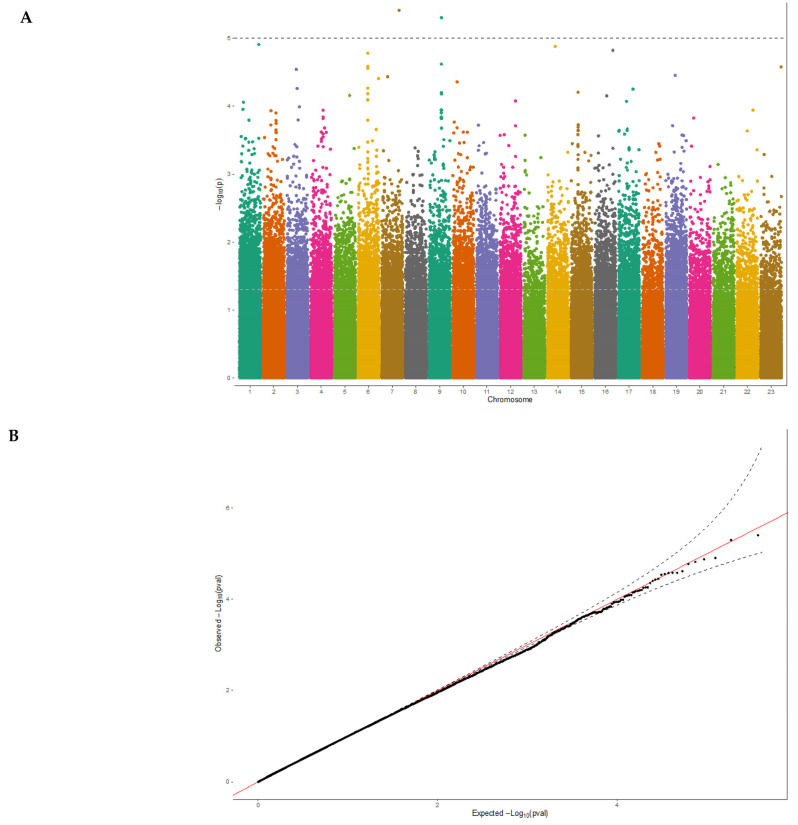
Manhattan plot and Q-Q plot of the ExWAS results for 25(OH)D levels. (**A**) Manhattan plot displays chromosomal positions of genetic variants (*x*-axis), and the –log10 *p*-value (*y*-axis) with a horizontal grey line represents the top signals of *p*-value < 1 × 10^−5^. The plot shows novel genomic regions on chromosome 7 and 9 that exceed the significance threshold of vitamin D ExWAS (*n* = 481,395 variants). (**B**) Q-Q plot displays a good fit between the observed –log10 *p*-values (*y*-axis) with expected –log10 *p*-values (*x*-axis), indicating that the ExWAS results are not biased and are consistent with the null hypothesis. The red line depicts the expected distribution of the *p*-values, while the black line represents the observed distribution. The black dashed lines represent the lower and upper limits of the 95% confidence interval.

The generalized linear mixed model was utilized to detect the genetic basis and potential causal genes of 25(OH)D levels in samples of the elderly Lebanese population who passed quality control criteria (*n* = 194 participants). In the model, we adjusted age, gender, and population PCs to account for any population stratification and relatedness, focusing on risk alleles with common frequency (MAF > 1%; *n* = 481,395). The Manhattan plot of the ExWAS revealed top SNP signals on chromosome 7 and chromosome 9 as potential risk loci for the circulating 25(OH)D levels at a *p*-value ≤ 1.0 × 10^−5^ (Figure 1A). These variants map to novel loci not previously associated with 25(OH)D levels. Our results showed no evidence of widespread inflation or significant population stratification (λ_GC_ = 1.002, stander error (SE) = 4.9 × 10^−6^), as shown in the Q-Q plot (Figure 1B).

**Figure 2:**
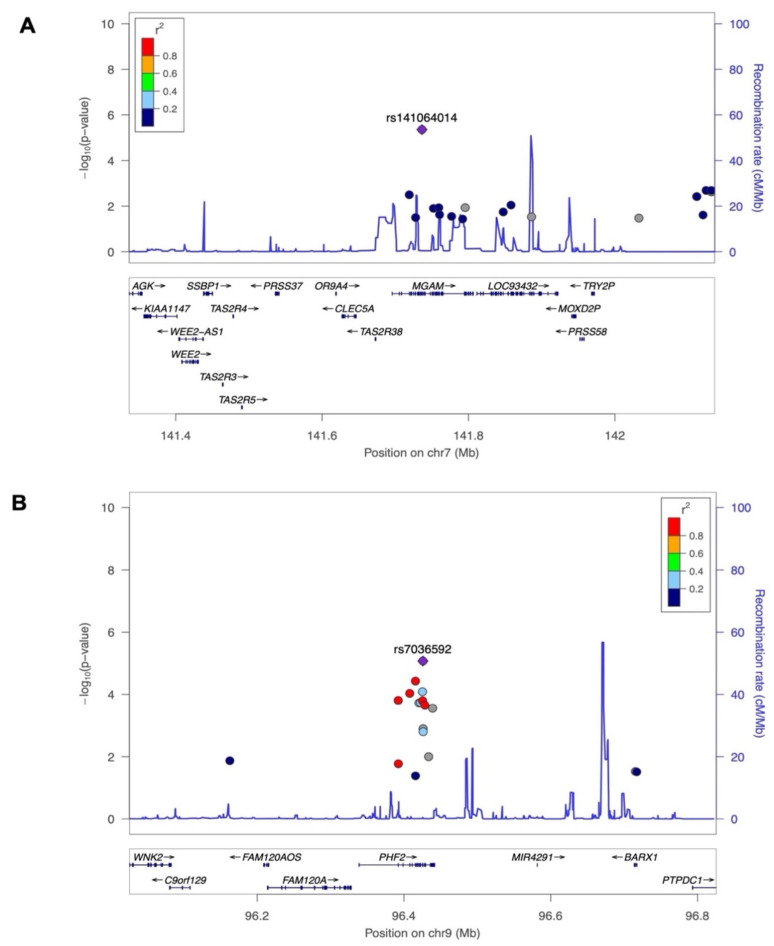
Genetic regions surrounding the top novel genetic variant that is associated with 25(OH)D levels using LocusZoom visualization tool. (**A**) the *MGAM* rs141064014 on chromosome 9; (**B**) the *PHF2* rs7036592 on chromosome 7 (top variant—shown in purple diamonds). On the Manhattan plot, the *p*-values are represented on the left vertical axis in log 10 scale, while the recombination rates are displayed as a blue line on the right vertical axis. The horizontal axis represents the chromosomal positions. In the bottom panel, the names and locations of genes are provided. The genes within the region are annotated and represented as arrows. The linkage disequilibrium relationships, indicated by the r^2^ values, between each SNP and the lead SNP are depicted using different colors.

The strongest association signal is at SNP rs141064014 (chromosome 7q34: 141736273: G>A) at a *p*-value of 4.40 × 10^−6^ (Table 2), in the intronic region of the maltase-glucoamylase (*MGAM*) gene. The pairwise LD assessment defined that the rs141064014 is not significantly correlated (r^2^ < 0.2) with any other SNPs within a genomic region spanning 250 Mb (Figure 2A). This indicates that rs141064014 is independent and shows no strong genetic correlation with nearby genetic markers over a considerable genomic distance. Another strong association is SNP rs7036592 (chromosome 9: 96425777: C>T) at a *p*-value of 8.43 × 10^−6^ in the plant homeodomain (PHD) finger 2 (*PHF2*) gene (Table 2), which is in LD with several other nearby SNPs in the same locus (Figure 2B). Variants suggestively associated with 25(OH)D levels with a *p*-value below 5 × 10^−4^ are shown in Table S1. Furthermore, we investigated the contribution of ExWAS SNPs to the variation in 25(OH)D levels within the Lebanese population. Through this analysis, we estimated that the heritability of 25(OH)D was approximately 29%, as determined by all the filtered SNPs.

### 3.3. Evaluation of Common Loci Replication

To evaluate the extent of replication, we performed a comparison between our findings to the largest vitamin D GWAS from the UK Biobank (GCST90000616). The GCST90000616 study, equivalent to our study, used an association analysis approach and rank-based inverse normalization to analyze 25(OH)D levels [12], enabling the comparison of effect weights for the identified loci in both cohorts. In the Lebanese cohort, we observed the replication of 7151 loci that showed significant association in the GCST90000616 study. Specifically, we replicated 58 variants that exhibited suggestive significance in the GCST90000616 study (Table S2).

A significant association was observed between the allele frequencies of the common SNPs, which exhibited a Pearson’s coefficient (R) of 0.62 (95% CI = 0.26 to 0.83) at a *p*-value of 0.0025. In addition, the correlation analysis of effect weights and directions for the common variants showed consistent directionality, with larger effect weights than the findings of the GCST90000616 (*n* = 12, R = 0.92, 95% CI = 0.74 to 0.98, *p*-value < 0.0001). The other variants (*n* = 46 variants) exhibited an opposite association direction as compared to GCST90000616, which could be influenced by genetic diversity within the populations, study design, and environmental factors that contribute to 25(OH)D levels.

### 3.4. Meta-Analysis of Vitamin D GWAS

We then conducted a meta-analysis to uncover potential new SNPs demonstrating significant genome-wide associations in the Lebanese dataset. This analysis involved combining our ExWAS results with summary statistics obtained from the largest European GWAS conducted by Revez JA et al. for vitamin D, consisting of 417,580 individuals. The replication GCST90000616 study has been previously described in detail [12]. Through our meta-analysis, we validated the replication of a missense variant rs2725405 (chromosome 17:79220224 C>G) at a *p*-value of 3.73 × 10^−8^, Beta = 0.0109. This variant is in the solute carrier family 38A10 (*SLC38A10*) gene that had been previously reported in the GWAS Catalog (*p*-value = 2 × 10^−8^, Beta = 0.0114) [12]. We further identified several variants in known loci (*n* = 70 variants), as presented in Table S3.

### 3.5. Analysis of Functional Variant Expression and Frequency

We compared the allele frequencies of the significant SNPs in the Lebanese samples with healthy individuals from the ALFA and gnomAD databases. The Lebanese elderly population exhibited a higher frequency of rs141064014, whereas the frequencies of rs7036592 and rs2725405 were lower compared to the European population (Table 3).

**Figure 3:**
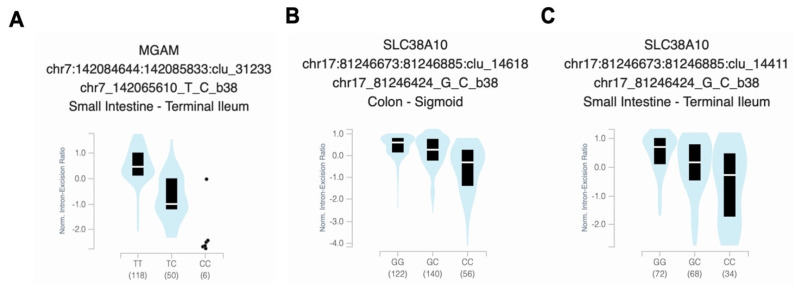
Relationship between the genotypes of vitamin D-associated SNPs and the gene expression enrichment. The bean plots display the normalized intron–excision ratio and their median (indicated by a white horizontal line) and interquartile range (represented by a black box) for (**A**) expression of *MGAM* rs141064014 in the small intestine with a significant *p*-value of 1.9 × 10^−21^; the expression of *SLC38A10* rs2725405 in (**B**) the colon with a significant *p*-value of 1.8 × 10^−48^; and (**C**) the small intestine with a significant association with *p*-value of 1.5 × 10^−23^. The data presented are derived from the GTEx database.

We performed a targeted tissue enrichment analysis using the GTEx map tool to gain insights into the potential functions of the top linked SNPs involved in vitamin D. We discovered notable correlations between particular alleles and decreased gene expression in the digestive tract and skin tissues when compared to other tissues. The homozygous risk allele “CC” and heterozygous “TC” of rs141064014 were significantly associated with reduced *MGAM* gene expression in the small intestine, with a *p*-value of 1.9 × 10^−21^ (Figure 3A). The presence of the “CC” risk allele of rs2725405 was associated with a significant reduction in *SLC38A10* gene expression in the colon (Figure 3C) and small intestine (Figure 3D), with *p*-values of 1.8 × 10^−48^ and 1.5 × 10^−23^, respectively. Finally, an enriched expression of the *PHF2* gene was detected in multiple tissues through the RNA-seq public resource ARCHS4, including chondrocytes of bone marrow and osteoblasts in bone tissues. The normalized gene expression, measured as transcript per million (nTPM), was approximately 9.7 (Figure S1).

### 3.6. Analysis of Polygenic Risk Score

**Figure 4:**
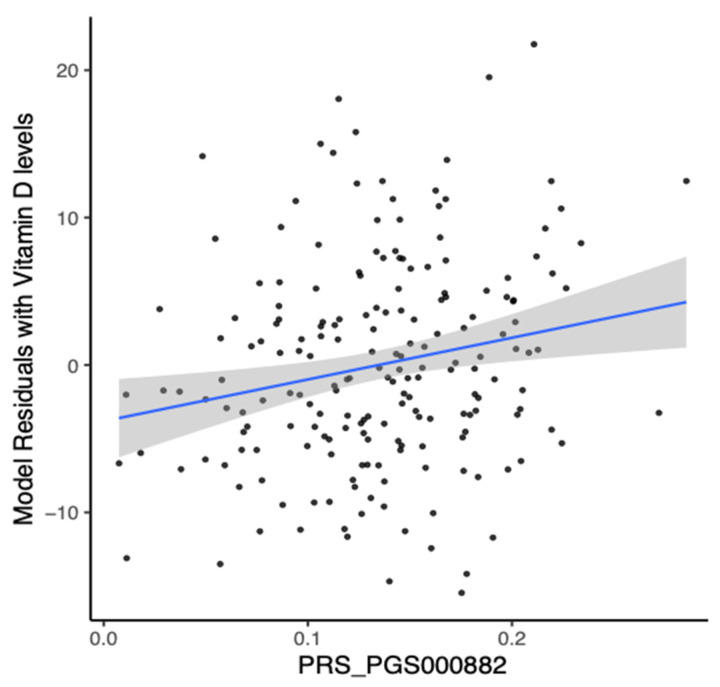
Performance of polygenic risk score derived from European population on the Lebanese individuals. Linear regression of the baseline levels of 25(OH)D and European-derived polygenic risk scores (PRS) (PGS000882: R = 0.2033, *p*-value = 0.0044). The blue line corresponds to the linear regression analysis, representing the best fit of the data.

We then assessed the performance of a European-derived PRS from panel PGS000882 [12]. Out of the 1,094,650 variants in this panel, we detected 41,736 in our whole-exome sequence data. The performance of these polygenic risk scores in predicting the levels of 25(OH)D in the Lebanese individuals is shown in Figure 4.

**Figure 5:**
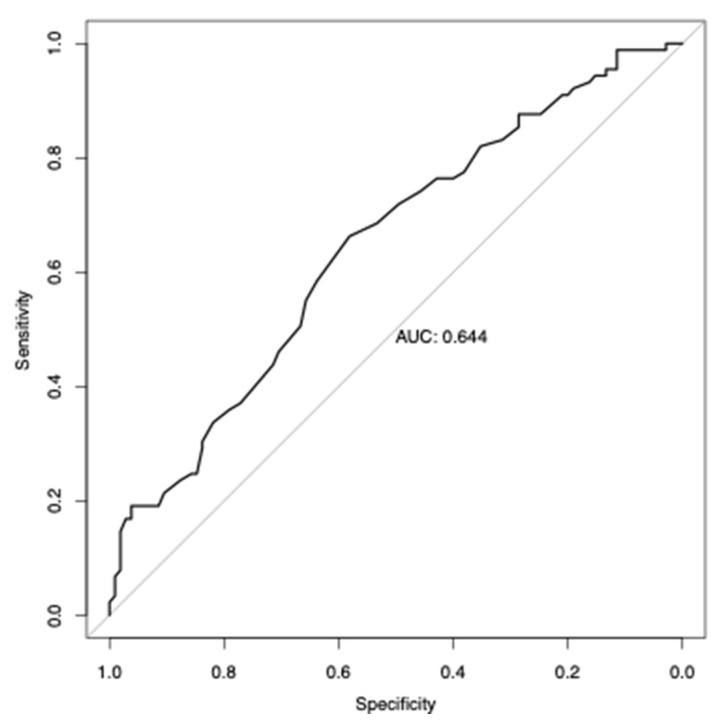
Performance of vitamin D deficiency prediction using European-derived PRS. The receiver operating characteristic (ROC) curve of the European-derived PRS was applied to evaluate the performance of predicting vitamin D deficiency (25(OH)D < 20 ng/mL) in Lebanese individuals.

The predictive performance of the European-derived PRS demonstrated lower results on the Lebanese cohort (R = 0.2033, 95% CI = 0.0643 to 0.3346, *p*-value = 0.0044) compared to the reported R values of 0.31 in the PGS000882 study. Despite this, the derived-PRS was able to efficiently predict the risk of vitamin D deficiency in Lebanese individuals, with an AUC of 0.644 (*p*-value = 0.0192, Odds Ratio = 1.6, 95% CI = 1.3 to 13.7) (Figure 5).

## 4. Discussion

Our study represents the first known attempt to detect common and new genetic SNPs that are linked to 25(OH)D levels in elderly individuals from the Middle East. Through a whole-exome analysis of 194 elderly Lebanese individuals, we discovered essential loci with suggestive evidence of an association with levels of vitamin D. The main findings of our study deviate from previous GWAS, where the most significant associated loci are located within four major genes, namely *GC*, *CYP2R1*, *CYP24A1*, and *DHCR7* [32]. Our research sheds light on previously unidentified genes that potentially contribute to the process of intestinal absorption of vitamin D. Importantly, this observation may be influenced by the specific characteristics of our cohort, which primarily consists of elderly individuals. Further validation of our results in independent populations, with an expanded sample size and utilizing a GWAS approach, would be advantageous. Indeed, such replication is the most reliable method to validate our findings.

In our study, the top genetic predisposition was the novel *MGAM* locus, rs141064014, with no indication of LD. The *MGAM* encodes the maltase-glucoamylase protein belonging to the glycoside hydrolase family 31 that breaks down complex carbohydrates in the small intestine [33]. In elderly individuals, reduced *MGAM* activity may exacerbate age-related changes in the digestive system, leading to reduced intestinal and hepatic enzymes, intestinal motility, and malabsorption of nutrients [34]. Our functional analyses showed that the “C” risk allele significantly reduces the intestinal expression of *MGAM*, which is highly elevated in our regional populations compared to other populations. These observations suggest a potential regulatory role in vitamin D absorption in the intestine among Middle Easterners, which requires further investigation.

We also identified a novel suggestive SNP, rs7036592, in the *PHF2* gene belonging to the Jumonji C family. *PHF2* is essential in several biological processes, such as the regulation of gene expression, different tissue functions, metabolism, and adipogenesis [35]. Interestingly, there is evidence that *PHF2* might be involved in regulating vitamin D metabolism and signaling [36]. *PHF2* has been shown to physically interact with and regulate the activity of *CYP27B1*, thereby affecting the synthesis of 1,25(OH)2D [37]. On the other hand, *PHF2* was found to involve in and enhance the transcriptional activity exhibited by the vitamin D receptor in osteoblasts, suggesting a potential role in regulating vitamin D-dependent gene expression [38]. Recent studies indicate that *PHF2* plays a crucial role in controlling the methylation pattern and subsequent expression of genes responsible for osteoblast differentiation in mice. Moreover, deleting *PHF2* in mice results in inadequate bone formation [39]. The high expression of *PHF2* in bone tissues suggests direct links with vitamin D in regulating osteoblast differentiation, which requires further investigation.

The heritability of vitamin D among Middle Easterners is currently unknown and requires further investigation. Previous GWAS has approximated the heritability of vitamin D to range from 7.5% to 16% among Europeans [10,13]. We found that the SNP-based heritability of vitamin D in the Lebanese group surpassed the estimation observed in UK Biobank participants, with a higher estimate of approximately 29%. This observation may be due to several factors, including study design, genetic diversity, and environmental exposures, which lead to increased heritability estimates [13]. Further investigation is needed to examine the underlying mechanisms driving these differences.

To ensure the validity and reliability of our findings, we examined data from the UK Biobank since the frequency and impact of alleles may differ across populations. Our analysis of this dataset enabled us to confirm several vitamin D-related variants identified in the GCST90000616 study, indicating the consistency of our results. Nonetheless, we acknowledge that differences in the genetic backgrounds of study populations and environmental exposures may have led to variants with opposing effect sizes. In order to better understand the reasons for opposing effect sizes and insufficient statistical power, further investigation may be needed, including studies in larger and more diverse populations.

To enhance the statistical significance of our observations, we merged our findings from Lebanese samples with data from the largest European GWAS [12]. Our meta-analysis has revealed additional SNPs related to 25(OH)D that were not considered in the GWAS catalog. Furthermore, we have confirmed the replication of multiple SNPs from the GCST90000616 study, including a missense variant, rs2725405, located in the *SLC38A10* gene. This gene is responsible for regulating protein transportation, synthesis, and cellular stress responses. In some cases, SLC38A10 protein can also mediate the intestinal transportation of some vitamins into the blood and lipid metabolism [40]. Notably, the expression of *SLC38A10* decreases in the presence of the “C” risk allele in the intestine and skin. The enrichment of *SLC38A10* in intestinal and skin tissues and regional populations, suggesting possible mechanisms in vitamin D absorption and metabolism. However, more research is needed to understand how it may impact vitamin D status.

While general recommendations of vitamin D deficiency management provide a foundation [4], personalized approaches hold value in identifying individuals at higher risk of vitamin D deficiency [7]. Various factors, such as genetic variations and environmental factors, can contribute to individual variations in vitamin D status and response to supplementation [6]. Therefore, personalized approaches can optimize interventions and address the specific needs of individuals of vitamin D deficiency. In our study, we derived PRS for predicting 25(OH)D levels in Lebanese individuals from European populations. The effectiveness of European-derived PRS in the Lebanese population was lower than that estimated in Europeans. This variation in performance might be due to factors such as variations in variant effect weights and frequencies of causal alleles across ethnicities, genetic heterogeneity, and the number of variants and participants utilized in the study [41]. These findings emphasize the need for a larger genome-wide association study tailored specifically for the Middle Eastern population to improve the performance of PRS estimation. Nevertheless, our polygenic risk score model for vitamin D demonstrated predictive ability in estimating vitamin D deficiency in the Lebanese cohort. The modest performance of the PRS in predicting 25(OH)D levels in individuals of European and Lebanese descent, with R values of 0.31 and 0.2033, respectively, may suggest the influence of nongenetic factors related to vitamin D deficiency, such as inadequate sunlight exposure and lifestyle/environmental factors. Therefore, the development of a more effective risk score tool for 25(OH)D levels may require the incorporation of such factors.

## 5. Conclusions

Overall, we explored the first suggestive evidence of an association between several loci and levels of 25(OH)D in an elderly Middle Eastern population through our ExWAS. Our study showed that ExWAS can more easily identify the genes and biological pathways associated with Mendelian phenotypes. The results of our PRS model may provide a new avenue for guiding the personalized treatment of vitamin D deficiency. However, further replications with increased sample sizes are necessary to confirm the potential of these findings and advance their application in the development of personalized medicine.

## Figures and Tables

**Table 1:** Mean characteristics of the enrolled subjects.

Characteristic	Male	Female	Total
Age (years)	72.7 (±5.50)	69.77 (±3.51)	71.13 (±4.82)
BMI (kg/m^2^)	28.69 (±3.35)	31.60 (±5.10)	30.24 (±4.60)
Serum 25(OH)D (ng/mL)	19.36 (±6.25)	20.83 (±7.93)	20.12 (±7.22)
Sample size	90 (46.4%)	104 (53.6%)	194

Descriptive statistics are presented as the mean (±SD) or as number (percentages, %) for Lebanese participant characteristics. Abbreviations: SD, standard deviation; BMI, body mass index.

**Table 2:** Variants of exome-wide association study for 25-hydroxyVitamin D levels.

SNP	Gene	HGVS ID	CHR	Position	A1	A2	Beta	SE (Beta)	*p*-Value
rs141064014	*MGAM*	NC_000007.13:g.141736273G>A	7	141736273	G	A	−2.38	0.52	4.4 × 10^−6^
rs7036592	*PHF2*	NC_000009.11:g.96425777C>T	9	96425777	C	T	−0.54	0.12	8.4 × 10^−6^

Statistical summary of the Lebanese ExWAS analysis using linear mixed models’ correction of principal population components, sex, and age with a *p*-value < 1 × 10^−5^. We used GRCh37/hg19 genome reference. Columns are SNP, single-nucleotide polymorphism rs ID; Gene, mapped genes affected by a variant from ANNOVAR; HGVS ID, Human Genome Variation Society sequence variant nomenclature descriptions from Ensembl; CHR, chromosome; A1, reference allele; BP, base pair; A2, alternative allele; Beta, variant effect size determined based on the allele A1; SE, the standard error for Beta; MAF, minor allele frequency determined based on the allele A1; *p*-value, *p*-value of ExWAS analysis. Abbreviations: *MGAM*, Maltase-glucoamylase; *PHF2*, PHD finger 2.

**Table 3:** Allele frequencies of the significant SNPs across different populations.

Populations	Frequency for rs141064014 in *MGAM*	Frequency for rs7036592 in *PHF2*	Frequency for rs2725405 in *SLC38A10*
Lebanese elderly population	0.0103	0.2408	0.4845
European population of ALFA	0.00794	0.39533	0.5729
Controls of gnomAD populations			
European	0.00634	0.3829	0.5468
East Asian	0.001	0.2136	0.3467
African	0.001	0.2783	0.9234
All populations	0.00553	0.3216	0.5084

Abbreviations: gnomAD, Genome Aggregation Database; ALFA, Allele Frequency Aggregator.

## Data Availability

All data produced during the study have been examined and incorporated into this published article or documented in the referenced data repositories. Publicly available software tools were used for all analyses, as indicated in the primary body of the text and Methods sections.

## Supplementary Materials

The following supporting information can be downloaded at: https://www.mdpi.com/article/10.3390/nu15143216/s1, Table S1: Single nucleotide polymorphisms identified in genome-wide analyses for circulating 25-hydroxyvitamin D concentrations in Lebanese samples; Table S2: Replication of loci identified by European GWAS (Revez JA et al., 2020) for vitamin D in the Lebanese GWAS; Table S3: Variants Discovered from a Meta-analysis of the Lebanese GWAS and GCST90000616 European study for Vitamin D Levels; Figure S1: Tissue Expression of PHF2 mRNA.
