# Supplementary material for: The Genetic Architecture of Vitamin D Deficiency among an Elderly Lebanese Middle Eastern Population: An Exome-Wide Association Study
Source: Nutrients. 2023 Jul 20;15(14):3216. doi: 10.3390/nu15143216 (PMC10384558; doi:10.3390/nu15143216)
Supplement: Supplementary file 1 [file nutrients-15-03216-s001.zip › Figure S1.pdf]

Supplementary information

The Genetic Architecture of Vitamin D Deficiency Among an Elderly Lebanese Middle Eastern Population: An Exome-Wide Association Study

Supplementary Figure

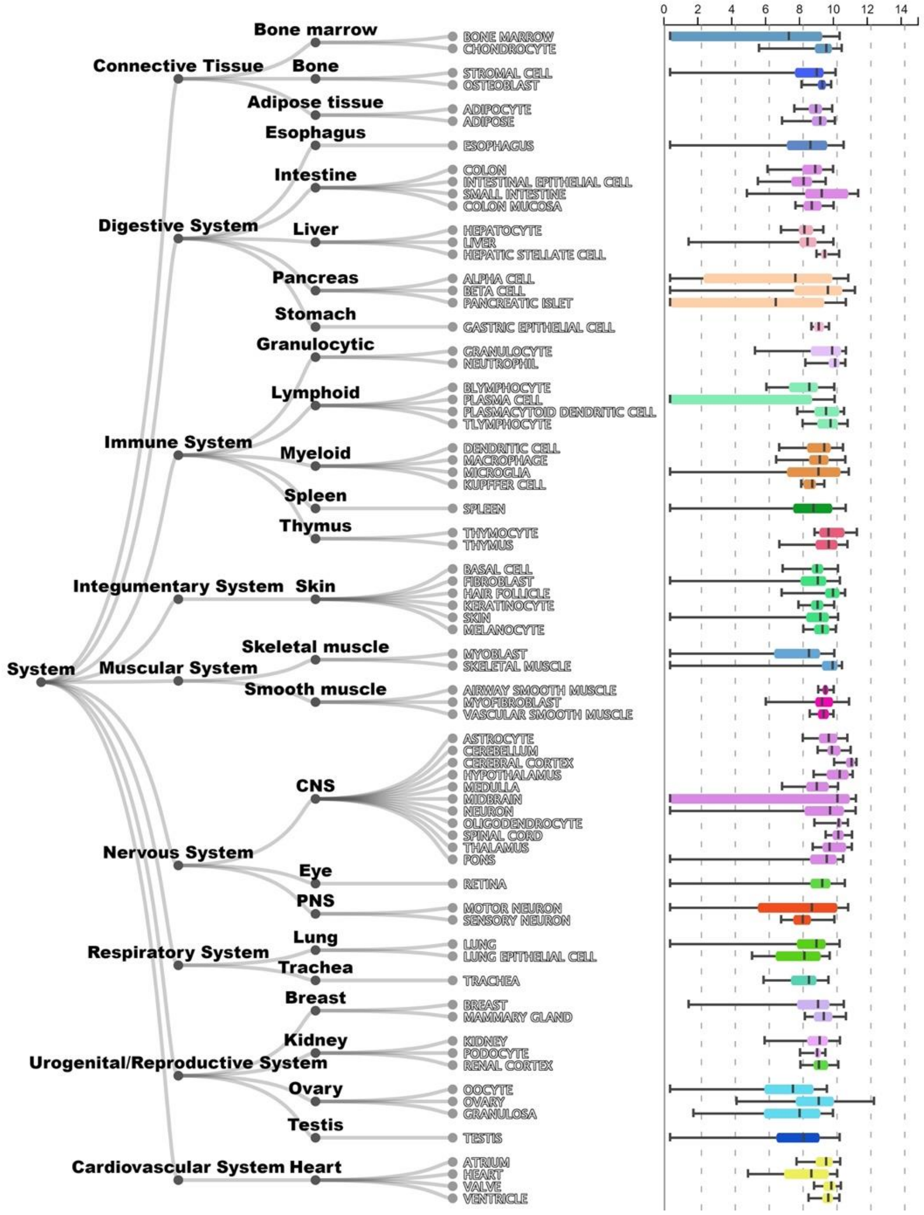

Figure S1. Tissue Expression of PHF2 mRNA.

The boxplot of normalized gene expression (nTPM) values obtained from the public RNAseq data analysis tool ARCHS4 shows PHF2's highest expression in the central nervous system (CNS), followed by chondrocyte of bone marrow and osteoblast of bone tissues.
